# High-resolution visualization of cervical spine anatomy using 3D DESS at 7-T MRI: a cohort study in asymptomatic volunteers

**DOI:** 10.1007/s00256-026-05356-3

**Published:** 2026-08-31

**Authors:** Christoph Germann, Veronika Zubler, Florian Wanivenhaus, Daniel Nanz, Reto Sutter

**Affiliations:** 1 Department of Radiology, Balgrist University Hospital, Forchstrasse 340, Zurich, CH-8008 Switzerland; 2 Department of Orthopedic Surgery, Balgrist University Hospital, Forchstrasse 340, CH-8008 Zurich, Switzerland; 3 University of Zurich, Zurich, Switzerland; 4 Swiss Center for Musculoskeletal Imaging, Balgrist Campus AG, Lengghalde 5, CH-8008 Zurich, Switzerland

**Keywords:** Magnetic resonance imaging, Spine, Dorsal root ganglia, Vertebral artery, Healthy volunteers

## Abstract

**Objective:**

To validate the feasibility of a three-dimensional dual-echo-steady-state (3D-DESS) sequence at 7-T MRI to reliably depict the detailed anatomy in the cervical spine.

**Materials and methods:**

Seventy-eight asymptomatic volunteers (age 36.4 ± 11.6 years) were imaged, using a 3D-DESS sequence at 7-T MRI. The course of the vertebral artery (VA) and the visualization of dorsal root ganglia (DRG) were analyzed by two fellowship-trained musculoskeletal radiologists. Additionally, degenerative changes were graded.

**Results:**

Uncovertebral arthrosis was present in 47.4% of participants; spinal canal stenosis was present in 16.1% (grade 1) and 2.6% (grade 2). Facet joint osteoarthritis at the most affected level was found in up to 12.8% (grade 1) and 3.8% (grade 2). Foraminal stenosis at the most affected level was seen in 10.3% (grade 1) and 3.8% (grade 2). Agreement was “almost perfect” for uncovertebral arthrosis (*κ* = 0.94) and spinal canal stenosis (*κ* = 0.82), “moderate” for facet joint osteoarthritis (*κ* = 0.56) and “substantial” for foraminal stenosis (*κ* = 0.68). Agreement was “perfect” regarding location of the VA (*κ* = 1.0), and almost perfect (*κ* = 0.93) for VA kinking. The agreement was “excellent” for distance between spinal cord and DRG (ICC = 0.94) and “good” for both width and height of DRG (ICC = 0.90 and 0.87, respectively). Contact between VA and DRG was present in almost all participants in levels C2/3 through C5/6 (94.9–100%).

**Conclusion:**

This study demonstrates the capability of a 3D-DESS sequence at 7-T MRI to reliably visualize the detailed anatomy of the cervical spine. Contact between VA and DRG is a relatively consistent finding in levels C2/3 through C5/6.

**Supplementary Information:**

The online version contains supplementary material available at https://doi.org/10.1007/s00256-026-05356-3.

## Introduction

7-T MRI can feature a substantially higher signal-to-noise (SNR) ratio than is available at lower field strengths, which can be invested in a higher spatial resolution with unchanged acquisition time or in a shorter acquisition time with similar image quality [1]. Since fast MR acquisitions have become more abundant at lower field strengths, the increase in SNR at 7-T MRI is predominantly used for higher spatial resolution and thus for the visualization of anatomical details or subtle lesions rather than to accelerate the sequences.

The downside of using 7-T MR systems—aside from the cost aspect—is the accentuation of various imaging artifacts compared to lower main magnetic field strengths (e.g., related to chemical shift or field inhomogeneities), lack of dedicated transmit-receive coils, and specific absorption rate (SAR) limitations due to higher energy deposition [2].

Gradient echo sequences, in particular three-dimensional (3D) dual-echo-steady-state (DESS) sequences have already been used in 7-T MRI of the musculoskeletal system and proven to provide high accuracy for depicting cartilage calcifications (“chondrocalcinosis”) in the knee and excellent visualization of cervical nerve rootlets [3, 4]. The 3D-DESS can provide a high isotropic spatial resolution and, particularly when using water-specific excitation, is less affected by the often strong chemical shift artifacts at 7-T, e.g., in 2D turbo-spin echo (TSE) imaging. Recently, it has been shown that 7-T DESS images allowed a more sensitive grading of cervical nerve root compression in patients with radiculopathy compared to clinical 3-T MRI and yielded a superior diagnostic confidence and image quality [5].

MRI of the cervical spine is among the most frequently performed examinations in both hospital and outpatient settings, constituting a critical component in the diagnostic evaluation of patients presenting with cervical radiculopathy. The predominant underlying pathology in these cases is spinal degeneration, including facet joint osteoarthritis, and degenerative disc disease (i.e., intervertebral osteochondrosis and intervertebral disc herniations). These conditions can result in cervical radiculopathy due to mechanical compression or irritation of the nerve roots, which may be managed conservatively with image-guided nerve root injections or surgically, most commonly via anterior cervical discectomy with fusion (ACDF). Although ACDF is generally considered a safe procedure, serious complications can arise, such as injury to the vertebral artery [6, 7]. Depending on the level of decompression, detailed preoperative imaging of the vertebral arteries—typically via computed tomography angiography (CTA)—may be required to delineate vascular anatomy and identify anatomical variants such as kinking or vascular loops, thereby reducing the risk of intraoperative vascular injury. This particularly applies to the upper cervical segments, where anatomical variants are most frequently encountered. However, CTA involves ionizing radiation and carries a small but significant risk of adverse reactions to iodinated contrast agents. While MR angiography (MRA) serves as an alternative, it is also associated with potential adverse effects, though these are encountered less frequently than those associated with iodinated contrast agents.

We hypothesize that 7-T MRI, in addition to offering high-resolution imaging of cervical spinal structures, may also allow for reliable visualization of the vertebral arteries and their anatomic relationship to adjacent neural elements.

This capability may, in selected cases, reduce the necessity for preoperative CTA (or MRA) when evaluation of vertebral artery anatomy is the only clinical concern.

Therefore, we analyzed the potential of a 3D-DESS sequence at 7-T MRI to reliably depict the detailed anatomy of the cervical spine, including degenerative changes, location, and size of small neural structures (DRG) and course of the vertebral arteries.

## Materials and methods

This prospective volunteer study was approved by the local ethics committee. Written informed consent was given by all participants prior to enrollment.

### Study population

Recruitment took place at Balgrist University Hospital over 13 months. The following inclusion criteria were applied: (1) age ≥ 18 years, (2) no present cervical neck pain/discomfort, (3) no prior injection and/or surgery of the cervical spine, and (4) no contraindication regarding MR imaging (e.g., cardiac pacemaker and severe claustrophobia). A total of 82 volunteers were enrolled. Due to poor image quality owing to major to severe motion artifacts (graded by both readers), four participants were excluded, resulting in a final cohort size of *n* = 78.

### Magnetic resonance imaging

Each participant underwent MR imaging of the cervical spine using an ultra-high-resolution 7-T MRI system (MAGNETOM Terra; Siemens Healthcare, Erlangen, Germany) with a dedicated 8-channel cervical spine surface array transmit-receive coil (Rapid Biomedical GmbH, Rimpar, Germany). A three-dimensional (3D) dual echo steady state (DESS) sequence of the cervical spine was acquired in the coronal plane. Detailed imaging parameters are listed in Table 1.

**Table 1:** DESS sequence parameter for 7-T MRI of the cervical spine

Parameter	DESS sequence
TR, *ms*	3.0
TE, *ms*	11.1
Excitation flip angle, *degrees*	20
FOV, *mm* (readout/phase/slab direction)	200/181/101
Voxel size, *mm*	0.63 × 0.63 × 0.63
Readout bandwidth, *Hz/pixel*	237
Parallel imaging acceleration factor	3
Acquisition orientation	Coronal
Acquisition time, *min:s*	06:38

*DESS* dual echo steady state, *FOV* field of view, *TE* echo time, *TR* repetition time

### Image evaluation

All MR images were transferred to state-of-the-art Picture Archiving & Communication System (PACS) workstations and evaluated separately by two board-certified fellowship-trained musculoskeletal radiologists (C. G. with 8 years and V. Z. with 16 years of experience). Multiplanar reconstructions (MPR) of the 3D-DESS datasets were used for the image analysis; this was performed in an independent and randomized fashion after removing all image annotations and participant-related data.

First, image quality due to artifacts (e.g., motion artifacts) was graded on a 5-point Likert scale as follows: grade 5 = “no artifacts,” grade 4 = “minimal artifacts,” grade 3 = “moderate artifacts, no impact expected on diagnostic value,” grade 2 = “major artifacts, critical impact on diagnostic value,” and 1 = “severe artifacts, no diagnostic value” [3]. The MRI dataset of each participant was incorporated in the further analysis if both readers rated the image quality at least as grade 3 (moderate artifacts, no impact expected on diagnostic value). Furthermore, each cervical spinal segment (C2-3 through C7-T1) was evaluated regarding the presence of uncovertebral arthrosis, i.e., anterior or posterior osteophytes involving the uncinate process, as well as for spinal canal stenosis according to the classification by Muhle et al.: grade 0 = normal, grade 1 = partial obliteration of the anterior or posterior subarachnoid space or of both, grade 2 = complete obliteration of the anterior or posterior subarachnoid space or of both, and grade 3 = cervical cord compression or displacement [8]. Additionally, each facet joint (C1-2 through C7-T1) was scrutinized for osteoarthritis and graded on a 4-point Likert scale adapted from Pathria et al. [9] and Weishaupt et al. [10]: Grade 0 = normal/no osteoarthritis, grade 1 = mild osteoarthritis (mild narrowing of the facet joint space and/or small osteophytes and/or mild hypertrophy of the articular process), grade 2 = moderate osteoarthritis (moderate narrowing of the facet joint space and/or moderate osteophytes and/or moderate hypertrophy of the articular process and/or mild subarticular bone erosions), and grade 3 = severe osteoarthritis (severe narrowing of the facet joint space and/or large osteophytes and/or severe hypertrophy of the articular process and/or severe subarticular bone erosions and/or subchondral cysts). Analysis of cervical foraminal stenosis of each segment was performed using the Park grading system [11]: grade 0 = no stenosis; grade 1 = mild perineural fat obliteration, < 50% of nerve root circumference, no morphological change of the nerve root; grade 2 = moderate perineural fat obliteration, ≥ 50% of nerve root circumference, and no morphological change of the nerve root; and grade 3 = collapsed nerve root with morphological change.

**Fig. 1:**
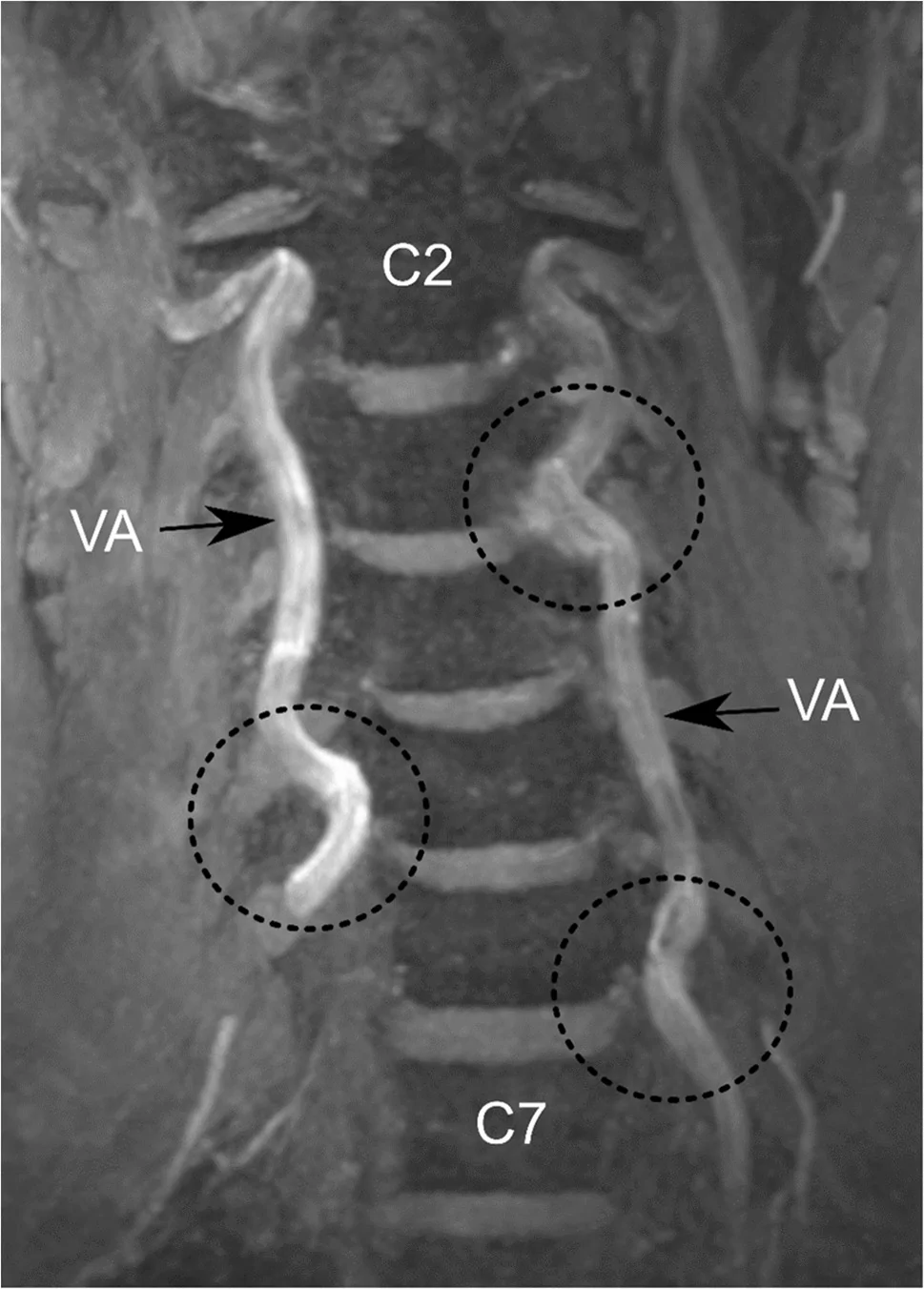
Kinking of vertebral arteries. Three-dimensional dual echo steady state (3D-DESS) sequence in the coronal plane depicting the cervical spine (vertebral bodies C2 through C7) of a 24-year-old female volunteer. Vertebral arteries (VA, arrow) are shown with clear elongated and arcuate course at the level C3/4 left, C5 right, and C6/7 left (dashed circles). VA, vertebral artery

**Fig. 2:**
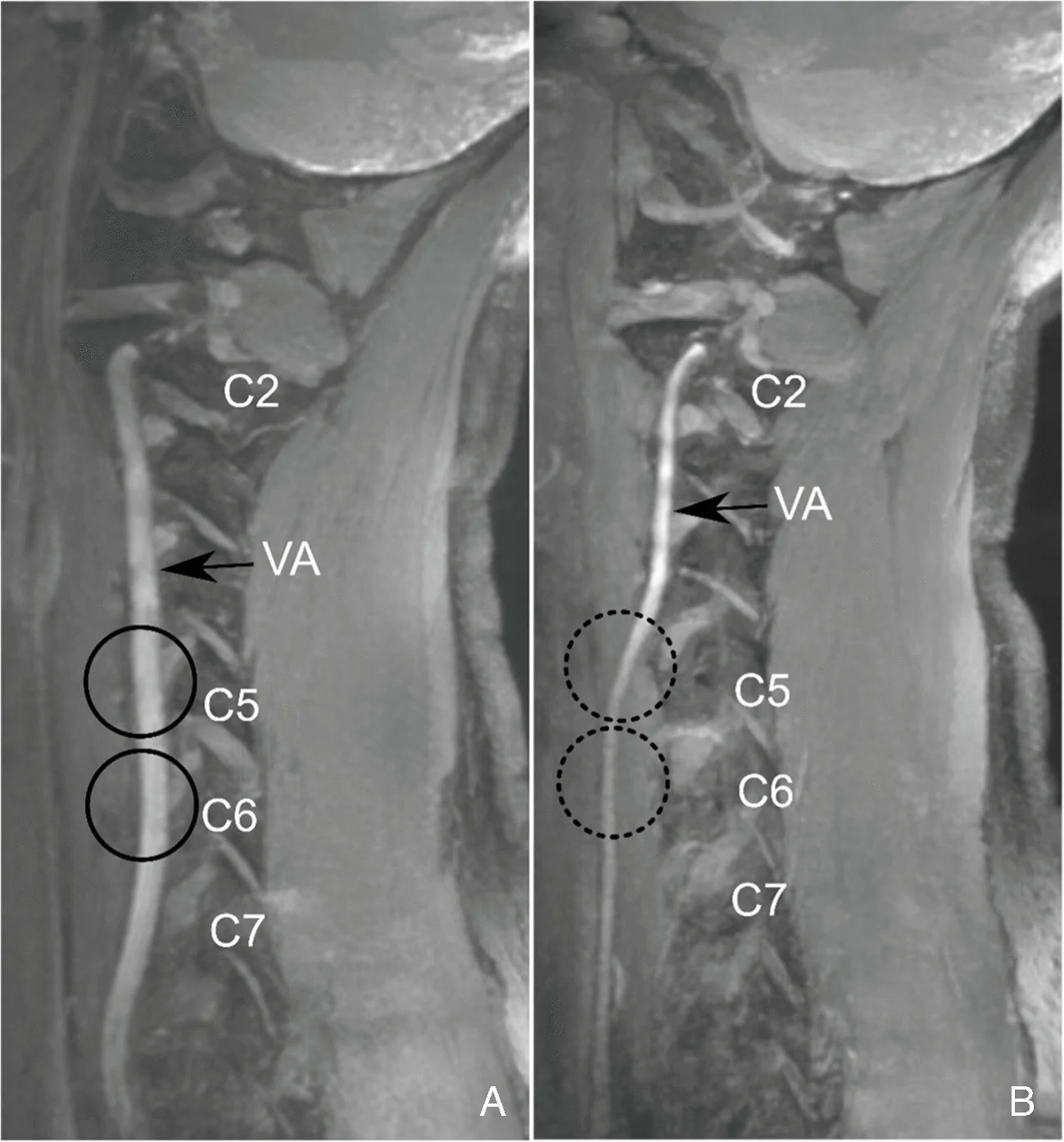
Variation in the course of the vertebral arteries. Sagittal MPR reconstructions of a three-dimensional dual echo steady state (3D-DESS) sequence depicting the cervical spine of a 25-year-old female volunteer. The course of the left (**A**) and right (**B**) vertebral artery (VA, arrow) is shown. At the level of the transverse foramen of vertebral body C5 and C6 the left-sided vertebral artery is located within the transverse foramen (circles) whereas the right vertebral artery is located outside of the transverse foramen in an anterior position (dashed circles). Note that there is a right dominance of the VA, with a larger diameter of the VA than on the left side. MPR, multiplanar reconstruction; VA, vertebral artery

**Fig. 3:**
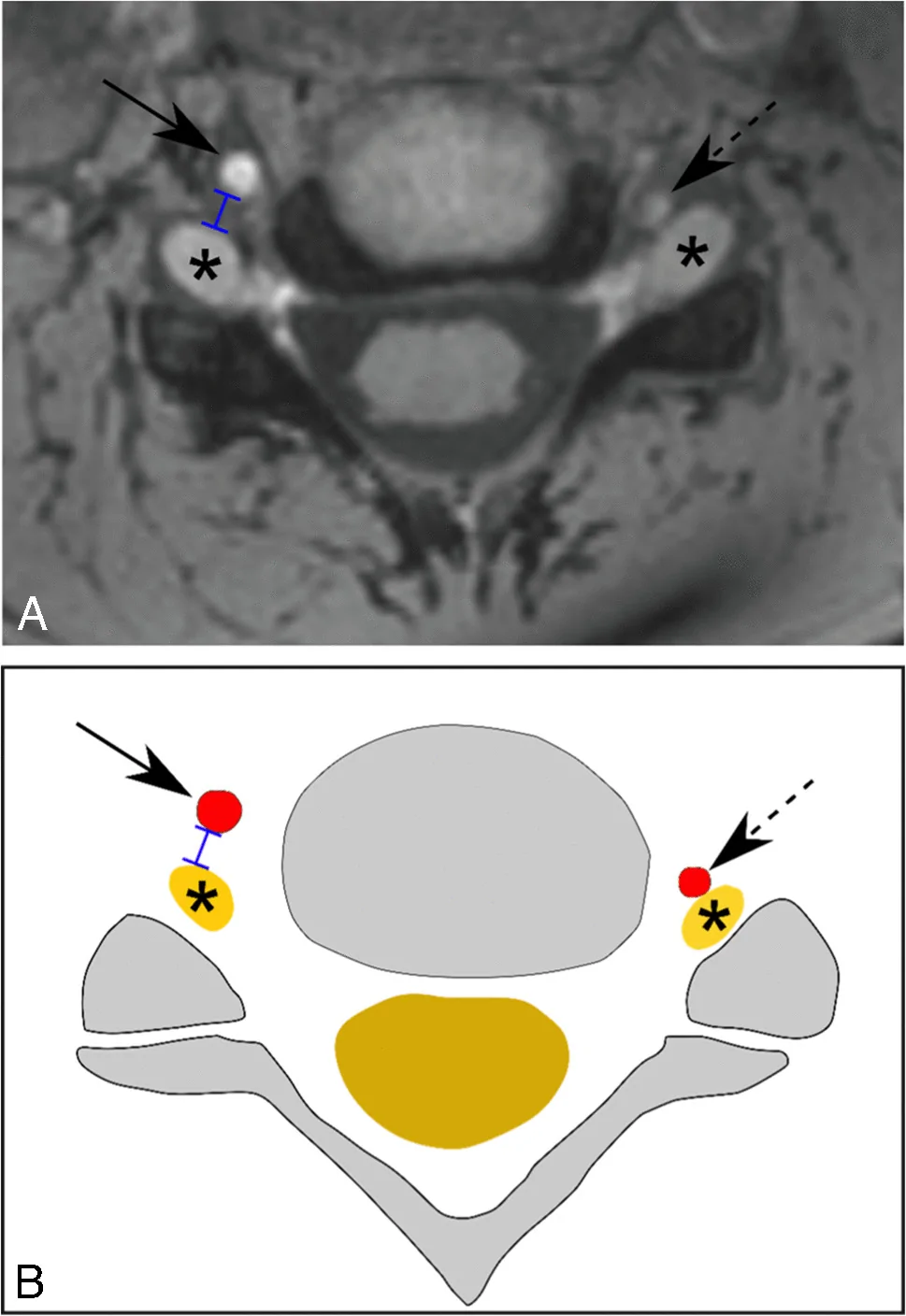
Relationship between vertebral artery and DRG. Axial MPR reconstruction of a three-dimensional dual echo steady state (3D-DESS) sequence depicting the cervical spine of a 52-year-old female volunteer at the C5/6-disc level (**A**) and corresponding graphic illustration (**B**). On the left side the vertebral artery (dashed arrow) is in contact with the DRG (asterisk), whereas on the right side there is a clearly discernible fat layer (blue line) in between the vertebral artery (arrow) and the DRG (asterisk). Note that there is a right dominance of the VA, with a larger diameter than on the left side. DRG, dorsal root ganglion; MPR, multiplanar reconstruction

Furthermore, the visualization of the vertebral artery was assessed. The course of the vertebral artery was scrutinized for variations: (a) kinking was defined as an arcuate course of the vertebral artery (Fig. 1); and (b) abnormal position in the V2-segment was defined as location outside the transverse foramen of vertebral body C6 through C2 (Fig. 2). Additionally, the anatomic relation of the vertebral artery to each dorsal root ganglion (DRG) C3 through C8 was analyzed in the axial plane as either “contact” or “no contact” between the two, whereas “no contact” signified a discernible fat layer between the vertebral artery and the ganglion (Fig. 3).

**Fig. 4:**
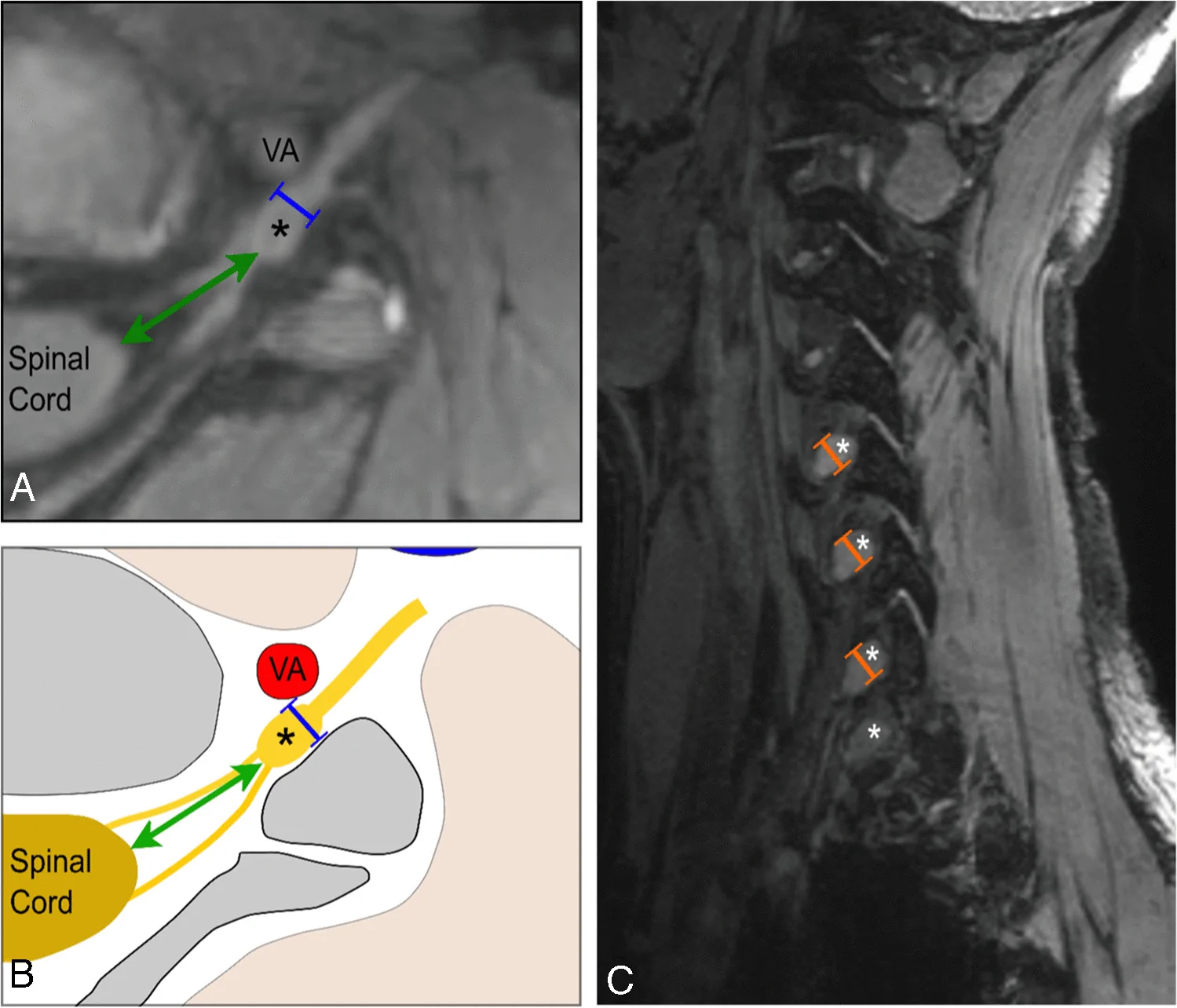
DRG Measurements. MPR-reconstructions of a three-dimensional dual echo steady state (3D-DESS) sequence in the axial (**A**, level C4-5) and sagittal (**C**) plane of the left cervical spine of a 23-year-old female volunteer. An axial image **B **corresponding to showing a graphic illustration **A **of the left-sided anatomy. The minimal distance between the spinal cord and the DRG (asterisk) is measured as shown by the green arrow. Additionally, the maximum width of the DRG is measured in the axial plane (blue line) and the maximum height of the DRG in the sagittal plane (orange line). DRG, dorsal root ganglion; MPR, multiplanar reconstruction; VA, vertebral artery

Finally, the visibility of the DRG was assessed: the minimal distance between the spinal cord and the dorsal root ganglion at each cervical spinal level was measured as illustrated in Fig. 3. Furthermore, the diameter of each dorsal root ganglion (C2 through C8) was measured in two dimensions: maximum width in the axial and sagittal plane, as shown in Fig. 4. Standardized reformatting planes were used for measurements: the axial plane was oriented parallel to the respective disc space, and the sagittal plane was oriented perpendicular to the axial plane.

### Statistical analysis

Statistical analysis was performed using SPSS (v25, IBM Corp., Somers, NY). Continuous data are presented as means with standard deviation and categorical data are presented as frequency and percentage. Interreader reliability was assessed using the intraclass correlation coefficient (ICC) for continuous variables, Cohen’s *κ* for qualitative binomial variables, and weighted *κ* for ordinal variables. ICCs were interpreted according to Landis and Koch [12], and *κ*-values according to Koo and Li [13]. Pearson’s correlation was used to test for any association between continuous demographic variables (age, height, and body mass index [BMI]) and measurements; point-biserial correlation was applied to evaluate the effect of sex on measurements. For correlation analysis the mean value of each measurement between reader 1 and reader 2 was used. A *p*-value < 0.05 was considered to represent statistical significance.

## Results

### Demographics

The mean age of participants was 36.4 ± 11.6 years. Forty-five of 78 subjects were female (57.7%); 33 were male (42.3%). The mean body height was 173.9 ± 9.6 cm; the mean body mass index was 23.7 ± 3.6.

#### Interreader reliability

The agreement between both readers was “almost perfect” for presence of uncovertebral arthrosis (*κ* = 0.94) and grading of spinal canal stenosis (*κ* = 0.82), “moderate” for grading of facet joint osteoarthritis (*κ* = 0.56), and “substantial” for grading foraminal stenosis (*κ* = 0.68). The interobserver agreement was “perfect” regarding location of the vertebral artery at each segment (*κ* = 1.0), and almost perfect (*κ* = 0.93) for detecting kinking of the vertebral artery.

The agreement between reader 1 and 2 regarding measurements was “excellent” for distance between spinal cord and DRG (ICC = 0.94) and “good” for both width and height of DRG (ICC = 0.90 and 0.87, respectively).

#### Degenerative changes of the cervical spine

The frequency of uncovertebral arthrosis and grading of spinal canal stenosis is given in Table 2 (data for readers 1 and 2). The grading of facet joint osteoarthritis and foraminal stenosis are given in Table 3 (data for reader 1) and in the Supplementary Material (data for readers 1 and 2). Uncovertebral arthrosis was present in up to 47.4% of participants, with the most frequently affected levels being C5/6 (47.7%), C6/7 (28.2%), and C4/5 (24.4%). Spinal canal stenosis was present in up to 16.7% (grade 1) and 2.6% (grade 2) of participants at level C5/6; the other cervical levels were rarely affected (0–6.4% for grade 1 and 0–1.3% for grade 2). Facet joint osteoarthritis was a rare finding in the asymptomatic study group: The most frequently affected levels were C6/7 (14.1% grade 1) and C5/6 (12.8% grade 1; 1.3% grade 2). Foraminal stenosis was encountered in up to 10.3% (grade 1) and 2.6% (grade 2) at level C6/7 and in up to 10.3% (grade 1) and 3.8% (grade 2) at level C5/6.

**Table 2:** Incidental findings: uncovertebral arthrosis and spinal canal stenosis

Level		**U**ncovertebral arthrosis Anterior/posterior **│** *n, (%)*	Spinal canal stenosis Grade 0/1/2/3** │** *n, (%)*
**C2/3**	*R1*	2 (2.6)/2 (2.6)	78 (100)/0 (0)/0 (0)/0 (0)
	*R2*	1 (1.3)/2 (2.6)	78 (100)/0 (0)/0 (0)/0 (0)
**C3/4**	*R1*	3 (3.8)/9 (11.5)	72 (92.3)/5 (6.4)/1 (1.3)/0 (0)
	*R2*	3 (3.8)/9 (11.5)	73 (93.6)/4 (5.1)/1 (1.3)/0 (0)
**C4/5**	*R1*	12 (15.4)/19 (24.4)	72 (92.3)/5 (6.4)/1 (1.3)/0 (0)
	*R2*	11 (14.1)/13 (16.7)	74 (94.9)/3 (3.8)/1 (1.3)/0 (0)
**C5/6**	*R1*	28 (35.9)/37 (47.4)	63 (80.8)/13 (16.7)/2 (2.6)/0 (0)
	*R2*	24 (30.8)/29 (37.2)	64 (82.1)/12 (15.4)/2 (2.6)/0 (0)
**C6/7**	*R1*	17 (21.8)/22 (28.2)	73 (93.6)/5 (6.4)/0 (0)/0 (0)
	*R2*	21 (26.9)/18 (23.1)	71 (91.0)/7 (9.0)/0 (0)/0 (0)
**C7/T1**	*R1*	2 (2.6)/4 (5.1)	78 (100)/0 (0)/0 (0)/0 (0)
	*R2*	4 (5.1)/4 (5.1)	78 (100)/0 (0)/0 (0)/0 (0)

The frequency of uncovertebral arthrosis (presence of anterior or posterior osteophytes at the uncinate process) and spinal canal stenosis (Grade 0, 1, 2 or 3 according to Muhle et al.) at each vertebral segment are shown for reader 1 (R1) and reader 2 (R2) for the entire cohort (n = 78). Data are presented as numbers (percentages in parenthesis)

**Table 3:** Incidental findings: Facet joint osteoarthritis and foraminal stenosis

Level		Facet joint osteoarthritis Grade 0/1/2/3** │** *n, (%)*	Foraminal stenosis Grade 0/1/2/3 **│** *n, (%)*
**C1/2**	L	78 (100)/0 (0)/0 (0)/0 (0)	78 (100)/0 (0)/0 (0)/0 (0)
	R	78 (100)/0 (0)/0 (0)/0 (0)	78 (100)/0 (0)/0 (0)/0 (0)
**C2/3**	L	71 (91.0)/5 (6.4)/1 (1.3)/1 (1.3)	76 (97.4)/1 (1.3)/1 (1.3)/0 (0)
	R	73 (93.6)/3 (3.8)/1 (1.3)/1 (1.3)	76 (97.4)/2 (2.6)/0 (0)/0 (0)
**C3/4**	L	71 (91.0)/3 (3.8)/3 (3.8)/1 (1.3)	66 (84.6)/8 (10.3)/3 (3.8)/1 (1.3)
	R	72 (92.3)/2 (2.6)/3 (3.8)/1 (1.3)	70 (89.7)/4 (5.1)/3 (3.8)/1 (1.3)
**C4/5**	L	73 (93.6)/3 (3.8)/2 (2.6)/0 (0)	65 (83.3)/8 (10.3)/3 (3.8)/2 (2.6)
	R	74 (94.9)/2 (2.6)/2 (2.6)/0 (0)	67 (85.9)/8 (10.3)/2 (2.6)/1 (1.3)
**C5/6**	L	67 (85.9)/10 (12.8)/1 (1.3)/0 (0)	66 (84.6)/6 (7.7)/3 (3.8)/3 (3.8)
	R	69 (88.5)/9 (11.5)/0 (0)/0 (0)	67 (85.9)/6 (7.7)/2 (2.6)/3 (3.8)
**C6/7**	L	67 (85.9)/11 (14.1)/0 (0)/0 (0)	68 (87.2)/8 (10.3)/2 (2.6)/0 (0)
	R	69 (88.5)/9 (11.5)/0 (0)/0 (0)	71 (91.0)/4 (5.1)/1 (1.3)/2 (2.6)
**C7/T1**	L	69 (88.5)/7 (9.0)/2 (2.6)/0 (0)	78 (100)/0 (0)/0 (0)/0 (0)
	R	70 (89.7)/7 (9.0)/1 (1.3)/0 (0)	78 (100)/0 (0)/0 (0)/0 (0)

The frequency of facet joint osteoarthritis (Grade 0, 1, 2 or 3) and foraminal stenosis (Grade 0, 1, 2, or 3 according to Park et al.) at each vertebral segment are shown for the entire cohort (*n* = 78). Numbers are given for reader 1, separately for each side (L and R). Data are presented as numbers (percentages in parenthesis). The complete data for both readers are given in the Supplementary Material

#### Vertebral artery (VA)

Deviation from the normal anatomy of the V2-segment (normal: course within the transverse foramen C2–C6) was observed in 8 of 78 subjects (10.3%) for the left as well as the right VA by both readers, meaning the course was atypical at the level of 16 transverse foramen in total: in 1 of these 16 events, the VA was outside the foramen at level C5, in 13 of 16 events the VA was located outside the foramen at level C6, and in 2 of 16 events the VA was located within the transverse foramen at level C7 (Fig. 2).

Kinking of the left VA in the V2-segment was detected in 6 of 78 volunteers (7.7%) for reader 1, and 8 of 78 volunteers (10.3%) for reader 2, whereas both readers found kinking of the right VA in 9 of 78 subjects (11.5%) (Fig. 1).

#### Dorsal root ganglia (DRG)

Measurement of the distance between the spinal cord and each DRG as well as the width and height of the DRG for both readers are listed in Table 4. The distance between the spinal cord and the DRG gradually increased from cranially to caudally, ranging from 7.8 ± 1.2 mm at level C1/2 up to 13.5 ± 1.9 mm at level C7/T1. The maximum width and height of DRG was smallest for level C3/4 (3.1 ± 0.4 mm and 3.6 ± 0.5 mm) with a tendency for a gradual slight increase going more caudally, showing the largest DRG at level C7/T1 (4.8 ± 0.6 and 5.3 ± 0.5).

**Table 4:** Measurements regarding dorsal root ganglia (DRG)

Level			Distance between spinal cord and DRG *Axial plane, in mm*	Width of DRG *Axial plane, in mm*	Height of DRG *Sagittal plane, in mm*
**C1/2**	*R1*	L R	7.8 ± 1.2 7.7 ± 1.3	3.9 ± 0.4 3.9 ± 0.4	4.4 ± 0.6 4.3 ± 0.5
	*R2*	L R	7.9 ± 1.3 7.6 ± 1.3	4.3 ± 0.5 4.3 ± 0.5	5.1 ± 0.7 5.0 ± 0.5
**C2/3**	*R1*	L R	9.5 ± 1.3 9.6 ± 1.6	3.5 ± 0.4 4.1 ± 0.5	4.1 ± 0.5 4.1 ± 0.5
	*R2*	L R	9.8 ± 1.4 9.6 ± 1.6	3.6 ± 0.4 4.2 ± 0.5	4.7 ± 0.5 4.7 ± 0.6
**C3/4**	*R1*	L R	9.5 ± 1.9 9.5 ± 1.9	3.1 ± 0.4 3.0 ± 0.4	3.6 ± 0.5 3.7 ± 0.4
	*R2*	L R	9.9 ± 1.7 9.5 ± 1.7	3.5 ± 0.6 3.6 ± 0.5	4.2 ± 0.6 4.3 ± 0.5
**C4/5**	*R1*	L R	11.1 ± 1.9 11.0 ± 2.0	3.3 ± 0.5 3.4 ± 0.5	4.1 ± 0.6 4.3 ± 0.5
	*R2*	L R	11.7 ± 1.9 11.2 ± 2.0	3.8 ± 0.7 4.0 ± 0.7	4.8 ± 0.6 5.0 ± 0.7
**C5/6**	*R1*	L R	12.0 ± 2.3 12.0 ± 2.0	4.0 ± 0.6 4.1 ± 0.5	5.0 ± 0.5 5.2 ± 0.5
	*R2*	L R	12.9 ± 2.2 12.4 ± 2.1	4.6 ± 0.7 4.9 ± 0.6	5.7 ± 0.7 6.0 ± 0.7
**C6/7**	*R1*	L R	12.8 ± 2.1 12.7 ± 2.1	4.6 ± 0.5 4.8 ± 0.5	5.3 ± 0.5 5.5 ± 0.5
	*R2*	L R	13.6 ± 2.0 13.4 ± 2.1	5.1 ± 0.6 5.5 ± 0.5	5.9 ± 0.7 6.2 ± 0.7
**C7/T1**	*R1*	L R	13.5 ± 1.9 13.3 ± 2.1	4.8 ± 0.6 4.9 ± 0.4	5.3 ± 0.5 5.4 ± 0.5
	*R2*	L R	14.0 ± 2.0 13.8 ± 2.1	5.3 ± 0.6 5.5 ± 0.6	6.1 ± 0.6 6.3 ± 0.7

Distance between the spinal cord and respective DRG and the width and height of each DRG; the measurements were performed on axial and sagittal images, respectively. Values are given as mean ± standard deviation. Data are shown separately for each cervical segment, each reader (R1 and R2) and for both sides (left: L; right: R)

*DRG* dorsal root ganglion

#### Relation between vertebral artery (VA) and dorsal root ganglia (DRG)

The frequencies of contact or no contact between VA and DRG at each level for both readers are presented in Table 5. Contact between VA and DRG was present in almost each participant in levels C2/3 through C5/6 (94.9–100%), in approximately one out of two participants in level C6/7 (53.8%) and almost never in level C7/T1 (2.6%).

**Table 5:** Contact between vertebral artery (VA) and dorsal root ganglia (DRG)

Level		Contact between vertebral artery and dorsal root ganglia Contact/no contact, *n* (%)	
		Left	Right
**C2/3**	*R1*	75 (96.2)/3 (3.8)	75 (96.2)/3 (3.8)
	*R2*	72 (92.3)/6 (6.7)	72 (92.3)/6 (6.7)
**C3/4**	*R1*	77 (98.7)/1 (1.3)	78 (100)/0 (0)
	*R2*	78 (100)/0 (0)	78 (100)/0 (0)
**C4/5**	*R1*	78 (100)/0 (0)	77 (98.7)/1 (1.3)
	*R2*	77 (98.7)/1 (1.3)	77 (98.7)/1 (1.3)
**C5/6**	*R1*	74 (94.9)/4 (5.1)	74 (94.9)/4 (5.1)
	*R2*	75 (96.2)/3 (3.8)	74 (94.9)/4 (5.1)
**C6/7**	*R1*	42 (53.8)/36 (46.2)	42 (53.8)/36 (46.2)
	*R2*	48 (61.5)/30 (38.5)	43 (55.1)/35 (44.9)
**C7/T1**	*R1*	2 (2.6)/76 (97.4)	2 (2.6)/76 (97.4)
	*R2*	0 (0)/78 (100)	1 (1.3)/77 (98.7)

The frequency of contact/no contact between the VA and DRG are shown for reader 1 (R1) and reader 2 (R2) for the entire cohort (*n* = 78). Data are presented as numbers (percentages in parenthesis)

*DRG* dorsal root ganglion, *VA* vertebral artery

#### Correlation between demographics and measurements of dorsal root ganglia (DRG)

The correlation analysis is shown in Table 6.

**Table 6:** Correlation analysis between demographics and measurements

	**Age** *p*-value (*r*)	**Body height** *p*-value (*r*)	**BMI** *p*-value (*r*)	**Sex** *p*-value (*r*)
Distance between spinal cord and DRG	**0.03** **(0.07)**	** < 0.001** **(0.28)**	0.47	** < 0.001** **(0.22)**
Width of DRG	0.66	**0.02** **(0.07)**	0.05	0.28
Height of DRG	0.88	0.63	0.29	0.35

*BMI* body mass index, *DRG* dorsal root ganglion, *r* Pearson’s correlation coefficient

Age (*r* = 0.07; *p* = 0.03) and body height (*r* = 0.29; *p* < 0.001) showed a significant positive correlation with the distance between the spinal cord and each DRG. Additionally, sex was significantly associated with the distance between the spinal cord and DRG (*r* = 0.22; *p* < 0.001): male sex was associated with a larger distance. In multivariable regression analysis, only age (*p* = 0.004) and body height (*p* < 0.001) maintained a significant positive correlation. In case of the variable age, however, the correlation has to be stated as negligible (*r* = 0.07), as it explains less than 1% of the observed variance.

No significant correlation was seen between BMI and distance between the spinal cord and the DRG (*p* = 0.47). A significant positive correlation was seen between body height and width of the DRG (r = 0.07, *p* = 0.02). No correlation was seen between each demographic variable and height of the DRG (all *p* > 0.28).

## Discussion

In this prospective study, we evaluated the feasibility of a three-dimensional dual-echo steady-state (3D-DESS) sequence at 7-Tesla MRI in healthy volunteers for high-resolution visualization of cervical spine anatomy. Our aim was to assess its capability to delineate degenerative changes, small neural structures—particularly the dorsal root ganglia (DRG)—and the vertebral arteries.

The depiction of anterior and posterior osteophytes/uncal process hypertrophy using 7-T MRI was excellent, as evidenced by near-perfect interreader agreement. Similarly, the assessment of spinal canal stenosis demonstrated high concordance between radiologists. Interobserver reliability for grading facet joint osteoarthritis and foraminal stenosis was moderate to substantial, aligning with previously reported literature values [14]. The moderate agreement in grading facet joint osteoarthritis may reflect limitations of gradient-echo imaging for detecting subtle cartilage and joint space changes in small facet joints, compared with conventional fluid-sensitive sequences. Additionally, the grading system used was developed for lumbar facets on conventional imaging [9] and cross-sectional imaging (CT and 1-T MRI) [10]; its unvalidated application to small cervical joints using 7-T DESS may introduce further variability.

Importantly, the visualization of the vertebral arteries was of high quality. We observed perfect agreement regarding the localization of the V2 segment and nearly perfect agreement concerning the presence of vascular kinking or elongation. The robust identification of the vertebral arteries was further supported by the high interreader agreement in detecting contact between the V2 segment and the DRG. This level of anatomical detail may be of critical relevance for surgical planning, especially at upper cervical levels where vascular variations, such as elongations or loops, are common, and pedicle screw placement occurs in close proximity to the artery. Accurate preoperative visualization of vertebral artery anatomy could significantly reduce the risk of vascular injury during decompression procedures [6, 15].

Contact between the vertebral artery and the DRG was a relatively consistent finding from C2/3 through C5/6 in this cohort of healthy, asymptomatic volunteers. This observation suggests that vertebral artery–DRG contact represents a normal anatomical configuration at these levels. In contrast, the prevalence of contact decreased markedly at C6/7 (53.8%) and C7/T1 (2.6%), likely reflecting the more lateral course of the vertebral artery along its V1 segment.

Our findings also highlight the potential of 7-T MRI for in vivo analysis of the DRG. Consistent with prior reports by Feuerriegel et al. [5], we confirm that 7-T MRI enables excellent visualization of DRG morphology. The DRG, housing cell bodies of primary sensory neurons, plays a crucial role in pain transmission [16]. Previous cadaveric work by Boyd-Clark et al. demonstrated an association between degenerative cervical changes and DRG morphology at segments C5–C7 [17], suggesting clinical relevance for detailed DRG assessment.

However, DRG are not reliably visualized using conventional standard MRI sequences at 1.5-T or 3-T. In contrast, our study and that of Feuerriegel et al. [5] show that 3D-DESS at 7-T MRI allows for precise measurement of DRG position and dimensions in vivo. We measured the shortest distance from the spinal cord to the DRG, achieving excellent interreader agreement. Our findings are consistent with anatomical studies indicating that this distance increases from rostral to caudal cervical segments [18].

Clinical relevance is underscored by work from Yabuki et al., who demonstrated that the response to fluoroscopy-guided nerve root injection (C6/C7) correlated with DRG position: a more distal DRG was associated with greater pain relief [18]. Thus, accurate DRG localization via 7-T MRI may have a prognostic value in therapeutic planning. Furthermore, we were able to reliably measure DRG width and height, observing the smallest dimensions at C4 and a progressive increase toward C8. Compared to Feuerriegel et al. [5], our measured DRG widths were approximately 1 mm smaller on average, likely due to methodological differences—our measurements were performed strictly in the axial plane (perpendicular to the respective disc space), whereas theirs were reconstructed perpendicular to the nerve axis. In addition, measurements obtained from reformatted images may be subject to variability related to partial-volume effects which may influence the delineation of anatomical margins and, consequently, the measured dimensions.

An important clinical question arising from these findings is whether changes in cervical DRG dimensions are associated with radiculopathy. We hypothesize that focal nerve root compression induces neuroinflammatory changes within the DRG, potentially resulting in measurable DRG hypertrophy on ultra-high-field 7-T MRI. Further studies are needed to assess whether DRG dimensions correlate with clinical symptoms in patients with cervical radiculopathy.

Additionally, to assess the potential advantage of ultra-high-field 7-T MRI over lower field strengths for detailed depiction of cervical spine anatomy and degenerative changes, studies directly comparing 7-T and 3-T MRI are warranted.

Our study has limitations. Due to severe motion artifacts, 4 of 82 subjects were excluded. These artifacts are a recognized limitation of ultra-high-field MRI, likely intensified by the system’s high sensitivity to motion and increased acoustic noise. Second, the relatively young cohort (mean age 36 years) is not representative of typical patients undergoing cervical spine MRI for neck pain, which likely accounts for the low prevalence of degenerative findings. However, the primary objective of this study was to demonstrate the technical feasibility of 7-T MRI for detailed anatomical assessment. Future studies should further investigate these findings in symptomatic populations with a higher prevalence of degenerative changes, as encountered in routine clinical practice. Additionally, 7-T MRI remains predominantly a research tool outside of brain and knee imaging. Technical advancements, including the development of dedicated cervical spine coils for clinical use, improved artifact correction strategies, and the validation and implementation of clinically established multi-contrast TSE sequences at 7-T MRI, are required for broader clinical adoption.

In conclusion, this study demonstrates that a 3D-DESS sequence at 7-T MRI enables reliable and detailed visualization of cervical spine anatomy, including degenerative changes, vertebral artery course, and DRG morphology. Contact between VA and DRG is a relatively consistent finding in levels C2/3 through C5/6. These findings suggest potential clinical applications for preoperative planning and the diagnostic evaluation of cervical radiculopathy.

## Supplementary Information

Below is the link to the electronic supplementary material.

ESM 1 (DOCX 34.4 KB)

## Data Availability

Not applicable.

## Abbreviations

3D-DESS: Three-dimensional dual echo steady state
DRG: Dorsal root ganglion
ICC: Intraclass correlation coefficient
MPR: Multiplanar reconstruction
TE: Echo time
TR: Repetition time
VA: Vertebral artery
