# Supplementary material for: High-resolution visualization of cervical spine anatomy using 3D DESS at 7-T MRI: a cohort study in asymptomatic volunteers
Source: Skeletal Radiol. 2026 Aug 31;55(12):3287–96. doi: 10.1007/s00256-026-05356-3 (PMC13669019; doi:10.1007/s00256-026-05356-3)
Supplement: Supplementary file 1 [file 256_2026_5356_MOESM1_ESM.docx]

**Supplementary Material**

| **Level** |  |  | **Facet joint osteoarthritis**  **Grade 0 / 1 / 2 / 3 │** *n, (%)* | **Foraminal stenosis**  **Grade 0 / 1 / 2 / 3 │** *n, (%)* |
| --- | --- | --- | --- | --- |
| **C1/2** | *R1* | L  R | 78 (100) / 0 (0) / 0 (0) / 0 (0)  78 (100) / 0 (0) / 0 (0) / 0 (0) | 78 (100) / 0 (0) / 0 (0) / 0 (0)  78 (100) / 0 (0) / 0 (0) / 0 (0) |
|  | *R2* | L  R | 78 (100) / 0 (0) / 0 (0) / 0 (0)  78 (100) / 0 (0) / 0 (0) / 0 (0) | 78 (100) / 0 (0) / 0 (0) / 0 (0)  78 (100) / 0 (0) / 0 (0) / 0 (0) |
| **C2/3** | *R1* | L  R | 71 (91.0) / 5 (6.4) / 1 (1.3) / 1 (1.3)  73 (93.6) / 3 (3.8) / 1 (1.3) / 1 (1.3) | 76 (97.4) / 1 (1.3) / 1 (1.3) / 0 (0)  76 (97.4) / 2 (2.6) / 0 (0) / (0) |
|  | *R2* | L  R | 73 (93.6) / 4 (5.1) / 1 (1.3) / 0 (0)  74 (94.9) / 3 (3.8) / 1 (1.3) / 0 (0) | 74 (94.9) / 3 (3.8) / 1 (1.3) / 0 (0)  76 (97.4) / 2 (2.6) / 0 (0) / (0) |
| **C3/4** | *R1* | L  R | 71 (91.0) / 3 (3.8) / 3 (3.8) / 1 (1.3)  72 (92.3) / 2 (2.6) / 3 (3.8) / 1 (1.3) | 66 (84.6) / 8 (10.3) / 3 (3.8) / 1 (1.3)  70 (89.7) / 4 (5.1) / 3 (3.8) / 1 (1.3) |
|  | *R2* | L  R | 74 (94.9) / 1 (1.3) / 2 (2.6) / 1 (1.3)  74 (94.9) / 2 (2.6) / 1 (1.3) / 1 (1.3) | 71 (91.0) / 3 (3.8) / 3 (3.8) / 1 (1.3)  74 (94.9) / 2 (2.6) / 2 (2.6) / 0 (0) |
| **C4/5** | *R1* | L  R | 73 (93.6) / 3 (3.8) / 2 (2.6) / 0 (0)  74 (94.9) / 2 (2.6) / 2 (2.6) / 0 (0) | 65 (83.3) / 8 (10.3) / 3 (3.8) / 2 (2.6)  67 (85.9) / 8 (10.3) / 2 (2.6) / 1 (1.3) |
|  | *R2* | L  R | 76 (97.4) / 2 (2.6) / 0 (0) / 0 (0)  76 (97.4) / 1 (1.3) / 1 (1.3) / 0 (0) | 73 (93.6) / 1 (1.3) / 3 (3.8) / 1 (1.3)  75 (96.2) / 1 (1.3) / 2 (2.6) / 0 (0) |
| **C5/6** | *R1* | L  R | 67 (85.9) / 10 (12.8) / 1 (1.3) / 0 (0)  69 (88.5) / 9 (11.5) / 0 (0) / 0 (0) | 66 (84.6) / 6 (7.7) / 3 (3.8) / 3 (3.8)  67 (85.9) / 6 (7.7) / 2 (2.6) / 3 (3.8) |
|  | *R2* | L  R | 74 (94.9) / 3 (3.8) / 1 (1.3) / 0 (0)  74 (94.9) / 4 (5.1) / 0 (0) / 0 (0) | 71 (91.0) / 2 (2.6) / 3 (3.8) / 2 (2.6)  71 (91.0) / 3 (3.8) / 2 (2.6) / 2 (2.6) |
| **C6/7** | *R1* | L  R | 67 (85.9) / 11 (14.1) / 0 (0) / 0 (0)  69 (88.5) / 9 (11.5) / 0 (0) / 0 (0) | 68 (87.2) / 8 (10.3) / 2 (2.6) / 0 (0)  71 (91.0) / 4 (5.1) / 1 (1.3) / 2 (2.6) |
|  | *R2* | L  R | 73 (93.6) / 5 (6.4) / 0 (0) / 0 (0)  75 (96.2) / 3 (3.8) / 0 (0) / 0 (0) | 73 (93.6) / 4 (5.1) / 1 (1.3) / 0 (0)  75 (96.2) / 1 (1.3) / 2 (2.6) / 0 (0) |
| **C7/T1** | *R1* | L  R | 69 (88.5) / 7 (9.0) / 2 (2.6) / 0 (0)  70 (89.7) / 7 (9.0) / 1 (1.3) / 0 (0) | 78 (100) / 0 (0) / 0 (0) / 0 (0)  78 (100) / 0 (0) / 0 (0) / 0 (0) |
|  | *R2* | L  R | 76 (97.4) / 2 (2.6) / 0 (0) / 0 (0)  75 (96.2) / 3 (3.8) / 0 (0) / 0 (0) | 78 (100) / 0 (0) / 0 (0) / 0 (0)  78 (100) / 0 (0) / 0 (0) / 0 (0) |

**Supplementary Material. Incidental findings: Facet joint osteoarthritis and foraminal stenosis.**

The frequency of facet joint osteoarthritis (Grade 0, 1, 2 or 3) and foraminal stenosis (Grade 0, 1, 2 or 3 according to Park et al.) at each vertebral segment are shown for the entire cohort (n=78). Numbers are given separately for reader 1 (R1) and reader 2 (R2) as well as for the left side (L) and right side (R). Data are presented as numbers (percentages in parenthesis).
